# Relocalization of DNA lesions to the nuclear pore complex

**DOI:** 10.1093/femsyr/fow095

**Published:** 2016-10-30

**Authors:** Catherine H. Freudenreich, Xiaofeng A. Su

**Affiliations:** Department of Biology, Tufts University, Medford, MA 02155, USA

**Keywords:** nuclear pore complex, recombination, DNA repair, collapsed replication fork, persistent DSB, eroded telomere

## Abstract

Early screens in yeast for mutations exhibiting sensitivity to DNA damage identified nuclear pore components, but their role in DNA repair was not well understood. Over the last decade, studies have revealed that several types of persistent DNA lesions relocate to either the nuclear pore complex (NPC) or nuclear envelope (NE). Of these two sites, the nuclear pore appears to be crucial for DNA repair of persistent double-strand breaks, eroded telomeres and sites of fork collapse at expanded CAG repeats. Using a combination of cell biological imaging techniques and yeast genetic assays for DNA repair, researchers have begun to understand both the how and why of lesion relocation to the NPC. Here we review the types of lesions that relocate to the NPC, mediators of relocation and the functional consequences of relocation understood to date. The emerging theme is that relocation to the NPC regulates recombination to influence repair pathway choice and provide a rescue mechanism for lesions or DNA structures that are resistant to repair.

## Teaser

The authors review how relocation of DNA lesions to the nuclear pore complex influences repair pathway choice and promotes genome stability.

**ABBREVIATIONS**
DSB: double strand break
HR: homologous recombination
BIR: break-induced replication
SSA: single strand annealing
MMEJ: microhomology-mediated end joining
NHEJ: non-homologous end joining
uSCR: unequal sister chromatid recombination
GCR: gross chromosomal rearrangement
ChIP: Chromatin Immunoprecipitation
HO: homothallic endonuclease (or cut site)
HU: hydroxyurea
MMS: methylmethanesulfonate
NPC: nuclear pore complex
NE: nuclear envelope

## INTRODUCTION

DNA repair must occur in the context of a crowded nucleus. For homologous recombination (HR), the additional challenge exists of finding the right homologous template for repair. This is facilitated by robust search mechanisms, including proteins such as Rad51 that mediate synapsis (Symington, Rothstein and Lisby 2014) and a regulated increase in mobility of the broken chromosome (Dion *et al.*^2012^; Mine-Hattab and Rothstein 2012; Dion and Gasser 2013). In late S or G2 phases, once a sister chromatid is available, it is the preferred template for repair, which is facilitated by proximity maintained by sister chromatid cohesion (Nasmyth and Haering 2009). Spontaneous damage that occurs in S phase is often repaired very quickly, in a manner of minutes (Lisby, Rothstein and Mortensen 2001). However, some types of damage appear to be more difficult to repair, and can persist, sometimes for hours, causing G2 arrest. In the last decade, it has been appreciated that some of these types of persistent lesions relocate to the nuclear periphery, and this relocation appears to play an important role in rescuing the repair process and facilitating its completion (see Lisby *et al.*^2010^; Nagai, Heun and Gasser 2010; Nagai, Davoodi and Gasser 2011; Geli and Lisby 2015 for other recent reviews). In this review, we will provide a brief summary of the current state of the field, focusing on the types of lesions known to relocate to the nuclear pore in *Saccharomyces cerevisiae*, and the possible roles of this event in facilitating or regulating DNA repair and fork restart.

## STRUCTURE OF THE YEAST NUCLEAR PERIPHERY

The nuclear periphery is the boundary between the nucleus and cytoplasm, which is composed of a nuclear envelope (NE) and many nuclear pores. In yeast, the NE is a bilayer membrane composed of inner and outer nuclear membranes. The yeast inner nuclear membrane contains at least 10 proteins, including Mps3, an essential Sad1-UNC-84 (SUN) domain protein (Lusk, Blobel and King 2007). Mps3 is required for duplication of the spindle pole body and telomere peripheral tethering (Jaspersen, Giddings and Winey 2002; Nishikawa *et al.*^2003^; Antoniacci, Kenna and Skibbens 2007; Bupp *et al.*^2007^). The non-essential N-terminal domain of Mps3 is not required for spindle pole body duplication and Mps3 integration to the envelope, but it is required for telomere and double-strand break (DSB) positioning (Bupp *et al.*^2007^; Oza *et al.*^2009^). In mammalian cells, there is also a protein network of intermediate filaments lining the inner nuclear membrane called lamins, which are important in DNA replication, repair and genome stability maintenance (Singh *et al.*^2013^; Gruenbaum and Foisner 2015).

**Figure 1.**
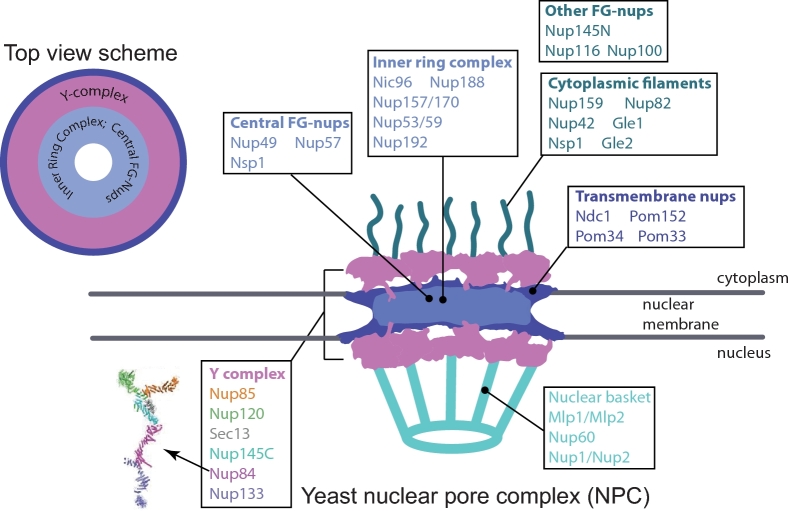
*Saccharomyces cerevisiae* NPC structure. The diagram is drawn based on Schwartz (2016); the composite high-resolution Y-shape complex structure is from Kelley *et al.* (2015). FG-nups are nucleoporins containing repetitive phenylalanine (F) and glycine (G) amino acid sequences.

Penetrating the NE are the nuclear pores, about 100–200 per cell in yeast, which provide a conduit between the nuclear and cytoplasmic compartments. The nuclear pore complex (NPC) is made up of at least 30 nucleoporins (usually named ‘nups’), and different groups of porins are further assembled into subcomplexes, which make up the basic building blocks of a nuclear pore (Schwartz 2016). The nuclear pore structure is highly conserved between yeast and more complex eukaryotic organisms. The largest nuclear pore subcomplex is the Y-shaped Nup84 complex (mammalian Nup107) (Fig. 1). It is a multiprotein complex with six core conserved members, and is the main component of the ring structure present on both nuclear and cytoplasmic sides of the pore (Fig. 1) (Schwartz 2016). There are several other subcomplexes located primarily internal to the ring structure formed by the Y complex (Fig. 1). The nuclear basket interacts with the pore ring and protrudes into the nucleoplasm; its components, Mlp1, Mlp2, Nup60, Nup1 and Nup2, are important in RNA export, gene gating, transcriptional regulation and DNA repair (Palancade *et al.*^2007^; Sood and Brickner 2014). The Y complex is also important for DNA repair, as mutation of Nup84, Nup120 or Nup133 causes hypersensitivity to DNA damaging agents, accumulation of spontaneous Rad52 foci and synthetic lethality with mutations that impair HR (Bennett *et al.*^2001^; Loeillet *et al.*^2005^; Palancade *et al.*^2007^; Nagai *et al.*^2008^). Nuclear pores have a highly conserved structure and function between human and yeast cells (Mekhail and Moazed 2010).

## RELOCATION OF DAMAGED DNA WITHIN THE NUCLEUS

Relocation of damaged DNA within the nucleus has been observed for several circumstances. Early on, it was discovered that DSBs within the ribosomal DNA (rDNA) relocate to a position outside the nucleolus during repair, and failure to do so leads to loss of rDNA repeats (Torres-Rosell *et al.*^2007^). Similarly, breaks in heterochromatic DNA move outside the locus to be repaired in Drosophila and mammalian cells (Chiolo *et al.*^2011^; Jakob *et al.*^2011^; Ryu *et al.*^2015^; Tsouroula *et al.*^2016^). Induction of a DSB in a yeast chromosome by the homothallic (HO) endonuclease does not lead to relocation. However, if the homologous donor sequences normally used for repair are removed or even moved to another chromosome, the broken chromosome relocates to the nuclear periphery (Nagai *et al.*^2008^; Kalocsay, Hiller and Jentsch 2009; Oza *et al.*^2009^). Similarly, a break that can heal by single strand annealing (SSA) with only 5 kb of resection does not relocate, but one that requires 30 kb of resection does (Oza *et al.*^2009^). These persistent or ‘difficult to repair’ DSBs relocate either to the nuclear pore via interaction with the Nup84 subcomplex or to the INM via interaction with Mps3. Recently, it was shown that the site of DSB relocation can depend on the cell cycle phase: DSB interaction with the Mps3 protein is restricted to S and G2 phases, whereas NPC interactions can occur in any cell cycle phase (Horigome *et al.*^2016^). The interaction site can also depend on DNA structure, as eroded telomeres in telomerase-deficient cells or induced subtelomeric DSBs relocalize to the NPC, though intact telomeres are normally tethered to the nuclear periphery by Mps3 (Therizols *et al.*^2006^; Bupp *et al.*^2007^; Khadaroo *et al.*^2009^; Schober *et al.*^2009^; Chung *et al.*^2015^). The lesions that have been found to relocate to the yeast nuclear pore or NE and the circumstances required are summarized in Tables 1 and 2, respectively.

**Table 1.** Interactions at the NPC and functional consequences.

Lesion type	NPC component associated with lesion	Cell cycle phase of association	Mediators (NPC interaction dependent on)	Function of relocalization	Reference
Persistent DSB (HO	Nup84 (ChIP)	G1/S/G2	Slx5/8	Increase survival	Nagai *et al.* (2008);
break, no donor for	Nup133 (ChIP)		Mec1/Tel1	Promote gene conversion	Kalocsay, Hiller and
repair)	Nic96 (ChIP)		Swr1	Promote ectopic BIR	Jentsch (2009); Oza
	Nup49 (Imaging^a, b^)		Mms21	Promote MMEJ	*et al.* (2009); Horigome
			Siz2	Suppress GCRs	*et al.* (2014, 2016)
			Smc5/6^e^		
Subtelomeric DSB	Nup84 (ChIP)	N/D	Kinesin14 (Cik1, Kar3)	Increase survival	Therizols *et al.* (2006);
			Cohibin (Lrs4, Csm1)	Promote end joining^f^	Chung *et al.* (2015)
			Swr1^e^	Promote Rad52-dependent BIR	
Eroded telomere	Nup49 (ChIP,	Senescing	Slx5/8	Relocalize to pores from NE	Khadaroo *et al.* (2009);
	(Imaging^a^)	cells	Siz1/Siz2	Promote Rad52-dependent	Churikov *et al.* (2016)
			Rad9/Rad24^e^	type II recombination	
Collapsed fork	Nup49 (Imaging^a, b^)	S	-	Increase survival	Nagai *et al.* (2008)
by HU + MMS^c^				Promote fork restart	
Collapsed fork at	Nup49 (Imaging^b^)	S	Nup84	Reduce repeat breakage and	Su *et al.* (2015)
CAG repeats^d^	Nup84 (ChIP)		Slx5/8	instability	
				Suppress Rad52-dependent HR	

a Colocalization of fluorescently tagged pore protein with the lesion in either wild-type or *nup133ΔN* mutant cells (which clusters NPCs to one side of the nucleus; Doye, Wepf and Hurt 1994).

b Preferential localization of the lesion at the periphery of the nucleus (zone 1) by zoning analysis.

c Induced collapsed fork by treatment with 0.2 M HU and 0.03% MMS.

d (CAG)_70_ or (CAG)_130_ repeat tracts.

e Mutant causes partial delocalization.

f Concluded to be NHEJ in Therizols *et al.* (2006), but a significant fraction (at least 40%) had what is now accepted as a MMEJ signature.

**Table 2.** Interactions at the NE and functional consequences.

Lesion type	NE component associated with lesion	Cell cycle phase of association	Mediators (NE interaction dependent on)	Function of tethering	Reference
Persistent DSB (HO break, no donor for repair)	Mps3 (ChIP, Imaging^a^) Heh2 (ChIP)	S/G2	Rad51, Rad52, Rad9/Rad24, H2A.Z^c^, Swr1, INO80 (Arp8) SMC5/6 (Nse5), Mms21, Rtt107	Delays HR repair Repress uSCR Repress HR with an ectopic donor Promote GCRs in NP mutants Recruit telomerase	Kalocsay, Hiller and Jentsch (2009); Oza *et al.* (2009); Horigome *et al.* (2014, 2016)
			Cdc13		
Slowly repaired DSB (30 kb resection required for SSA)	Mps3 (ChIP)	N/D	N/D	Unclear (repair is dependent on Rad52, partially on Nup84)	Oza *et al.* (2009); Chung *et al.* (2015)
Repairable DSB (HO break, ectopic donor on different chromosome)	Mps3 (ChIP^b^, Imaging^a^)	N/D	N/D	Suppress HR with an ectopic donor	Oza *et al.* (2009); Horigome *et al.* (2014)

a Preferential localization of the lesion at the periphery of the nucleus (zone 1) is lost in the *mps3ΔN* mutant.

b Repairable DSBs do not show a zone 1 increase by imaging or bind to Mps3 by ChIP (Nagai *et al.*^2008^; Oza *et al.*^2009^).

c H2A.Z is encoded by the *HTZ1* gene; NE interaction is also lost in *htz1-K126R, K133R* non-sumoylatable mutants (Kalocsay, Hiller and Jentsch 2009) or the *htz1ΔM6* mutant that doesn't bind SWR-C, but retains its Mps3 inner nuclear membrane localization function (Gardner *et al.*^2011^; Horigome *et al.*^2014^).

Besides DSBs, and eroded telomeres, collapsed replication forks relocalize to the nuclear periphery, specifically the nuclear pore (Nagai *et al.*^2008^; Su *et al.*^2015^). When replication forks stall, multiple outcomes are possible. A fork stalled by depletion of nucleotides (for example by, treatment with hydroxyurea (HU)) or encountering a barrier can retain an intact replisome for at least some period of time. In yeast, the replisome is still associated with forks stalled for 1 h in HU, and forks can restart replication if the HU is removed (Cobb *et al.*^2005^). These HU stalled forks do not relocate to the nuclear pore (Nagai *et al.*^2008^; Su *et al.*^2015^). Forks that encounter a nick (induced by the Flp-nick system) also remain in the interior of the nucleus (Dion *et al.*^2012^). On the other hand, prolonged incubation in HU or pretreatment with the DNA alkylating agent methylmethane sulfonate (MMS) before release into HU, conditions that induce repair foci, triggers relocation (Nagai *et al.*^2008^). Recently, we found that forks that encounter expanded CAG repeats of 70–130 units transiently move to the nuclear periphery in late S phase, interacting with Nup84 before disengaging by G2 phase (Su *et al.*^2015^). Chromosomes with longer repeats relocate more frequently. Repeats expanded to this range accumulate Rad52 foci and cause a checkpoint response that is mild but measurable in a significant proportion of cells, with a more severe response in a subset of cells (Gellon *et al.*^2011^; Sundararajan and Freudenreich 2011). The level and transient nature of the relocation to the NPC argues that the perinuclear attachment is part of a normal cellular response to forks stalled at the expanded CAG tract, which can form secondary structures that interfere with replication and repair (Su *et al.*^2015^; Usdin, House and Freudenreich 2015).

**Figure 2.**
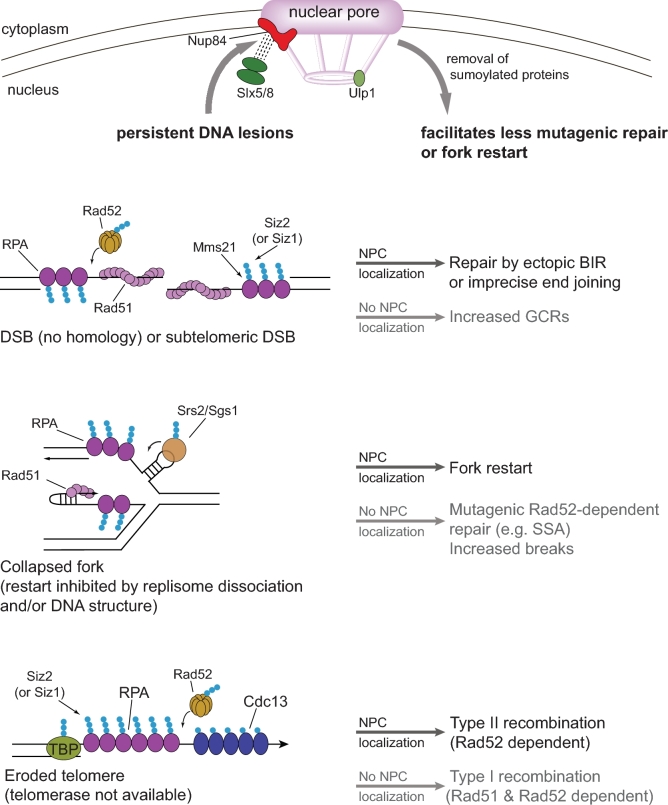
Summary of lesions found to relocate to the nuclear pore, some sumoylated proteins expected to bind them and observed functional outcomes of relocation. At top is a model for the function of relocation of persistent DNA lesions to the nuclear pore. The dashed line between Slx5/Slx8 STUbL and the Y-shaped Nup84 complex indicates interaction between Nup84 and Slx8 shown by Nagai *et al.* (2008). The isopeptidase Ulp1 that can cleave SUMO from modified proteins interacts with the nuclear basket. Three types of persistent DNA lesions that have been studied are illustrated at left. Only a few key proteins are shown; other proteins that have been shown to interact with these lesions and/or play a role in mediating interaction with the nuclear pore or NE are listed in Tables 1 and 2. The small blue circles represent SUMO (either mono-SUMO or poly-SUMO chains). TBP stands for telomere binding proteins. On the right are listed the known outcomes of interaction with the nuclear pore for each type of lesion (black arrows), or alternative outcomes that occur when NPC interaction is defective (gray arrows).

An interesting question is, what is the state of the fork that provokes relocation? A collapsed fork is generally agreed to be one that has lost replication competence, which in most cases probably also includes loss of a functional replisome (see Neelsen and Lopes 2015 for review). In the above cases, the stalled fork may be unable to restart effectively due to excessive damaged bases or hairpin structures. A collapsed fork may be converted to a broken fork, for example, by the action of structure-specific nucleases acting on the stalled fork. However, this step may not be the key factor in determining relocation since the broken fork caused by replication encountering a nick does not provoke the response, and pore association of the CAG tract actually prevents repeat fragility. Another factor that could play a role is fork reversal. If leading and lagging strand synthesis becomes uncoupled, the fork can regress, perhaps aided by annealing of the two nascent strands, to form a four-way junction or chicken foot structure visible by electron microscopy (see Neelsen and Lopes 2015 for review). In yeast, fork reversal has been shown to occur at uncoupled forks with extensive single-strand DNA (ssDNA) regions, for example, in cells that are checkpoint defective (Lopes *et al.*^2001^; Sogo, Lopes and Foiani 2002), or undergoing topological stress (Ray Chaudhuri *et al.*^2012^). In addition, there is experimental evidence for fork reversal occurring naturally in unperturbed cells at both CAG and GAA repeats, using direct visualization of replication intermediates by 2D gel electrophoresis and electron microscopy (Fouche *et al.*^2006^; Kerrest *et al.*^2009^; Follonier *et al.*^2013^). Although fork reversal can be a transient response to replication stress that facilitates lesion bypass, it could also lead to ‘stuck’ intermediates that are resistant to fork remodeling and restart (Fig. 2; collapsed fork).

## MECHANISMS OF DNA DAMAGE RELOCATION

Once one of the persistent lesions discussed above forms, how does it trigger relocation to the nuclear pore? A common theme emerging is that sumoylation pathways play a key role in mediating the interaction (Table 1). SUMO (small ubiquitin-like modifier) modifies lysine residues of its target substrates. Covalent addition of SUMO (encoded by the *SMT3* gene in yeast) is achieved by SUMO ligases: in yeast the only E2 ligase is Ubc9, and there are three E3 ligases that provide specificity, Siz1, Siz2 and Mms21, all part of the PIAS family also found in mammalian cells (Sarangi and Zhao 2015). SUMO can be removed by the SUMO-specific isopeptidases Ulp1 and Ulp2; Ulp1 is located at the nuclear pore (Li and Hochstrasser 1999; Zhao, Wu and Blobel 2004) (Fig. 2). Many DNA repair proteins are sumoylated, and several proteomic studies have identified sumoylated proteins present under normal or DNA damaging conditions (see for example Cremona *et al.*^2012^; Psakhye and Jentsch 2012; Albuquerque *et al.*^2013^; Thu *et al.*^2016^). Typically, levels of sumoylated proteins are very low as only a small percentage is modified. There is evidence that sumoylation is an ‘on-site’ modification that only occurs when the target protein is bound to DNA damage (Sarangi and Zhao 2015). Some notable DNA repair proteins known to be sumoylated in both yeast and human cells and present at breaks or collapsed forks are Rad52, RPA (Rfa1-3), Mre11, Sgs1, Srs2, Cdc13 and Smc5/6, but there are many others (Galanty *et al.*^2009^; Cremona *et al.*^2012^; Psakhye and Jentsch 2012; Sarangi and Zhao 2015; Thu *et al.*^2016^).

The Slx5/8 complex and its human homolog RNF4 are SUMO-targeted Ubiquitin Ligases (STUbLs) that contain multiple SUMO-interacting motifs (SIM domains). The Slx5/8 complex provides an intriguing link between on-site sumoylation at DNA lesions and relocation to the NPC, as it interacts with Nup84, and mediates relocation of each of the lesions shown to interact with the nuclear pore tested to date, including an expanded CAG tract, eroded telomere and persistent DSB (Table 1) (Nagai *et al.*^2008^; Su *et al.*^2015^; Churikov *et al.*^2016^; Horigome *et al.*^2016^). Both Ulp1 and the proteasome (which targets ubiquitylated substrates) are also located at the NPC, which could facilitate either desumoylation or degradation of sumoylated proteins that relocate there (Geli and Lisby 2015). The Slx5/8 homolog RNF4 mediates chromatin removal of MDC1, RPA, FANCI and FANCD2, which are important sensors of stalled forks and replication stress (Galanty *et al.*^2012^; Gibbs-Seymour *et al.*^2015^). Similarly, deletion or mutation of the *Saccharomyces cerevisiae* or *Schizosaccharomyce pombe SLX5* or *SLX8* genes render cells hypersensitive to HU (Mullen *et al.*^2001^; Zhang *et al.*^2006^; Prudden *et al.*^2007^).

One model for how Slx5/8-mediated chromosome relocation could work (Fig. 2) is that upon DNA damage, sumoylated repair proteins accumulate at the lesion site, which would bind Slx5/8 via the SIM domains in Slx5 (Xie *et al.*^2007^; Horigome *et al.*^2016^). Slx5/8 would then mediate interaction with the pore by its ability to bind Nup84 (Nagai *et al.*^2008^). Recent work from the Gasser lab showed that monosumoylation by Mms21, a component of Smc5/6, a cohesion-like complex, is sufficient for relocation of a DSB to Mps3, but that polysumoylation by Siz2 is needed for relocation to the NPC in G1 phase. Eroded telomeres were shown by Smt3 chromatin immunoprecipitation (ChIP) to accumulate SUMO-modified proteins coincident with cell senescence (when telomeres are short) as well as with Slx8 binding (Churikov *et al.*^2016^). In addition, telomere relocation to the NPC was impaired by deletion of Slx8, Siz1 or Siz2, though *slx8Δ* had the greatest effect. Lastly, RPA is polysumoylated during senescence, and physically interacts with Slx5/8 (Chung and Zhao 2015; Churikov *et al.*^2016^), making it a prime target for mediating the interactions necessary for relocation to the NPC (Fig. 2). Whether this model will apply to collapsed forks relocating in S phase is less clear. Slx5/8 was important for CAG tract relocation, although the defect was not as great as in a *nup84Δ* (Su *et al.*^2015^). However, Nup84 has additional functions beyond interaction with Slx5/8, for example, it is required for stabilization of Ulp1 and its association with the NPC (Palancade *et al.*^2007^), and this defect could contribute to the delocalization of the CAG tract. Also, Slx5/8 is only partially required for DSB–pore interaction in S phase, even though it is fully required in G1 (Horigome *et al.*^2016^).

There is also evidence for involvement of other mechanisms in relocation to the NPC in addition to SUMO interactions. Deletion of several other genes was shown to disrupt NPC localization of DSBs or eroded telomeres, including Swr1, Mec1/Tel1 and Rad9/Rad24 (Table 1). One thing all these proteins have in common is that they have been shown to be important in DSB mobility that occurs after break induction (Dion *et al.*^2012^; Mine-Hattab and Rothstein 2012; Horigome *et al.*^2014^). Interestingly, the checkpoint proteins Mec1 and Rad53 also play a role in stabilizing stalled replication forks that encounter active transcription, which is hypothesized to occur by release of transcribing genes from basket nucleoporins to reduce DNA topological tension (Bermejo *et al.*^2011^). It is not known whether these functions are related to their role in NPC localization. Another player is the Smc5/6 complex. The Nse5 subunit, which facilitates Smc5/6 recruitment to sites of replication stress (Cook, Hochstrasser and Kerscher 2009; Bustard *et al.*^2012^), can interact directly with Slx5, and ablation of this interaction reduced interaction with Mps3, though it had a more minor role in pore association (Horigome *et al.*^2016^). The Slx5-Smc5/6 interaction is conserved in *S. pombe* (Prudden *et al.*^2007^), and the Smc5/6 complex, along with another STUbL interacting complex RENi (Rad60, Esc2, Nip45) is necessary for relocation of heterchromatic DSBs in flies (Chiolo *et al.*^2011^; Ryu *et al.*^2015^). Even more proteins have been implicated in DSB localization to Mps3, including the telomere binding protein Cdc13 (Table 2). Recently, it was found that a subtelomeric DSB moves from the NE location normally occupied by telomeres to the NPC, and this movement was dependent on cohibin, a telomere tethering complex, kinesin14, a motor protein complex and α-tubulin (Chung *et al.*^2015^). The authors propose that an active microtubule-motor process moves the damaged telomere between sites. Thus, there may be multiple overlapping systems that recruit damaged DNA to the nuclear periphery, and determine the sites of interaction.

## FUNCTIONAL CONSEQUENCES OF DAMAGE RELOCATION

An important question is, what is the purpose of relocation to the nuclear pore or NE? This question has been addressed by monitoring DNA repair or telomere addition in mutants defective in relocation. Another strategy that has been employed is to artificially tether DNA to the pore and determine whether repair outcome changes using a genetic reporter assay. Functions of perinuclear tethering at each type of lesion that have been surmised from these approaches are summarized in Tables 1 and 2. Though there are still many questions, some themes are emerging. One fairly consistent result is that tethering at the NE by Mps3 generally suppresses HR, indicating that it is likely a repair repressive environment (Table 2). In contrast, the NPC appears to be a more permissive environment, promoting several types of repair (Table 1). However, there appear to be subtleties about which types of repair are promoted or suppressed by NPC localization, which may vary somewhat by lesion type or cell cycle phase. The most consistent theme is that a persistent lesion, which has failed to repair by a preferred or conservative mechanism, may relocate to the NPC to allow an alternative mechanism of repair to occur. This generally promotes survival and promotes repair, but at the expense of genome stability (Fig. 2).

For example, for a DSB, the preferred pathway of repair would usually be Rad51-dependent HR with a sister chromatid, or non-homologous end joining (NHEJ), which leads to minimal mutations (Symington and Gautier 2011). However, alternate pathways available are repair from an ectopic site, which can result in loss of heterozygosity, or microhomology-mediated end joining (MMEJ), which often results in large deletions or insertions (Sfeir and Symington 2015; Rodgers and McVey 2016). For a one-ended break, such as would occur at an eroded telomere or collapsed fork, break-induced replication using another chromosome as the template (ectopic BIR) would be an alternative rescue mechanism if telomere addition and fork restart on the same chromosome were not functioning.

The data available to date suggest that NPC localization is facilitating just such ‘alternate’ repair choices that may be mutagenic but are a preferred alternative to death or gross chromosomal rearrangements (GCRs) (Fig. 2). For example, for a persistent DSB, it was found that ectopic BIR and MMEJ were reduced in *slx8* or *nup84* mutants, and cell death and GCRs increased (Nagai *et al.*^2008^; Horigome *et al.*^2016^). For a subtelomeric DSB, survivors usually used BIR to repair, which was reduced in mutants with defective NPC localization, and artificial tethering to the pore hyperactivated BIR (Chung *et al.*^2015^). Pore-dependent BIR events assayed with a variety of reporters were all highly dependent on Rad52 and Pol32, two proteins previously established to be necessary for ectopic BIR (Anand, Lovett and Haber 2013; Chung *et al.*^2015^). Eroded telomeres can be rescued by either type I or type II recombination, with type II being more prominent in survivors; however, NPC mutants exhibit severely reduced numbers of type II recombinants (Churikov *et al.*^2016^). Indeed, tethering of an eroded telomere to Nup60 resulted in a significant increase in type II recombination events, providing direct evidence for a role for the NPC location in stimulating this event (Churikov *et al.*^2016^). Recently, Nup60 was found to be both monoubiquitylated and sumoylated; ubiquitylated Nup60 interacts with the Nup84 complex to tether it to the NPC and contributes to the cellular response to DNA damage (Nino *et al.*^2016^).

Consistently, a ubiquitin-deficient mutant of Nup60 favors the maintenance of type I survivors (Nino *et al.*^2016^).

The NPC-dependent events that occur at a collapsed fork are less clear. In common with other lesions, relocation has a positive effect on survival, as it prevents breaks at the expanded CAG repeat (since repeat-associated chromosome end loss events are increased in the absence of Slx5/8 or Nup84) (Su *et al.*^2015^). In addition, CAG expansions and contractions are increased in *slx5/8* or *nup84* mutants, and instability was caused by a process with greater dependence on Rad52 than Rad51 (Fig. 2). Thus, the data suggest that NPC association inhibited a mutagenic Rad52-dependent pathway or pathways that are prone to causing repeat instability, such as mutagenic SSA or another recombination pathway that can occur by Rad52-dependent annealing. Alternatively, it may be that for a collapsed fork, the preferred alternative pathway facilitated by NPC association is HR-dependent fork restart, but that some type of ‘release’ on recombination is required for restart to occur efficiently at a ‘stuck’ fork.

Rad52 sumoylation is induced upon DNA damage by MMS, and sumoylated Rad52 is ubiquitylated by Slx5/8 (Sacher *et al.*^2006^; Burgess *et al.*^2007^; Xie *et al.*^2007^). Interestingly, in the case of collapsed forks, degradation of a Rad52-Smt3 fusion protein (mimicking monosumoylation at the C-terminus) occurred coincident with NPC association, in a manner dependent on Slx8 (Su *et al.*^2015^). Rad52 sumoylation regulates repair efficiency of several different repair pathways. For example, in cells containing a mutant Rad52 protein lacking sumoylation sites, a shift from SSA to gene conversion, an increase in direct and inverted repeat recombination and a reduction in BIR and other types of interchromosomal events were observed (Altmannova *et al.*^2010^; Silva *et al.*^2016^). In addition, the Rad52 paralog Rad59 has two sites of sumoylation, and Rad52 and Rad59 sumoylation appear to act in synergy, with double mutants showing more pronounced phenotypes compared to the singles. The authors propose that the non-sumoylated forms of these proteins promote Rad51-dependent intrachromosomal types of recombination, while events that require more robust annealing (such as BIR or interchromosomal recombination) are promoted by the sumoylated forms (Silva *et al.*^2016^). Thus, regulation of Rad52 sumoylation by NPC relocation is one mechanism that could influence repair outcomes.

## CONCLUSIONS

These studies in yeast have elucidated an important role for movement of chromosomes between different nuclear compartments to facilitate DNA repair and regulate pathway choice. Several available tools in yeast have allowed this field to advance, including genetic knockouts, the ability to mark chromosomes and proteins fluorescently to follow their nuclear location and mobility, inducible systems for particular types of DNA damage, ease of cell cycle analysis and tools to tether particular chromosome regions to protein complexes. In addition, a wealth of assays for different repair pathways and outcomes (unequal sister chromatid recombination, BIR, NHEJ, MMEJ, SSA, GCRs, telomere rescue pathways, etc.) have allowed researchers to correlate localization to different nuclear compartments with repair outcomes. Importantly, the findings from yeast appear to be highly relevant to understanding similar events in other types of eukaryotic cells, though differences are also evident. For example, results from the Chiolo lab found that in *Drosophila* cells, heterochromatic DSBs move to the nuclear periphery before undergoing HR repair, a protective mechanism that prevents ectopic recombination (Chiolo *et al.*^2011^; Ryu *et al.*^2015^). This relocalization also depends on nuclear pore and inner nuclear membrane proteins that anchor repair sites to the nuclear periphery (Ryu *et al.*^2015^). Furthermore, it appears that this DSB targeting also relies on SUMO and SUMO E3 ligases (Ryu *et al.*^2015^). There is also compartmentalization of DNA repair in mammalian cells (see Lemaitre and Soutoglou 2015 for review). Similar to the situation in yeast, the nuclear lamina appears to be a repressive environment for HR (with NHEJ or MMEJ preferred), whereas the NPC is permissive for HR (Lemaitre *et al.*^2014^). However, in this case, movement between the lamina and the NPC was not detected. On the other hand, Lamin A/C depletion causes sensitivity to agents that stall forks (HU, interstrand cross-linking agents) and defective fork restart (Singh *et al.*^2013^), so it appears that regulation of both DSB repair and fork restart may be occurring at perinuclear domains in mammalian cells, as in yeast. The association between nuclear periphery components and fork restart in human cells, combined with the parallels between yeast Slx5/8 and human RNF4 (which interacts with sensors of stalled forks), suggests that relocation of collapsed forks may also be important to prevent genome instability in human cells. Expanded triplet repeats are a physiologically relevant site of fork stalling that have been shown to perturb replication in human cells (Voineagu *et al.*^2009^; Cleary *et al.*^2010^; Liu and Leffak 2012), and thus could be dependent on such a relocation process to prevent further expansions or chromosome breaks at the repeat. Also, mutations in Lamin A cause a degenerative disease marked by premature aging, Hutchinson-Gilford progeria syndrome, and patient cells exhibit accumulation of DNA damage, defects in DNA repair, genomic instability and telomere shortening (Gonzalo, Kreienkamp and Askjaer 2016). Mutations in human nucleoporin genes have been reported in a variety of inherited diseases and cancers, but links to defects in DNA repair have not yet been made (Nofrini, Di Giacomo and Mecucci 2016). Overall, these examples indicate that the connection between DNA repair, fork restart and perinuclear location is likely to be conserved across species, even if some details may prove to be different. The data gained from studies in yeast over the last decade, using various types of induced DNA damage along with informative genetic assays and imaging approaches, provide a framework for understanding the interplay between nuclear repositioning and regulation of DNA repair.

## FUNDING

The researchers were funded by Tufts University and NIH grant P01GM105473 to CHF.

***Conflict of interest.*** None declared.
